# Nucleated red blood cell distribution in critically ill patients with acute pancreatitis: a retrospective cohort study

**DOI:** 10.1186/s12876-024-03444-z

**Published:** 2024-10-07

**Authors:** Huan-qin Liu, Guan-qun Wang, Cheng-shuang Zhang, Xia Wang, Ji-kui Shi, Feng Qu, Hang Ruan

**Affiliations:** 1 Department of Critical-care Medicine, Jining NO.1 People’s Hospital, Jining, 272000 Shandong Province China; 2 Department of Critical-care Medicine, Tongji Hospital, Tongji Medical College, Huazhong University of Science and Technology, Wuhan, 430030 Hubei Province China; 3 Department of Emergency Medicine, Tongji Hospital, Tongji Medical College, Huazhong University of Science and Technology, Wuhan, 430030 Hubei Province China

**Keywords:** Critical care, Cohort study, Nucleated red blood cell, Acute Pancreatitis, Risk factors

## Abstract

**Objectives:**

This study examined the potential association between nucleated red blood cell (NRBC) levels and mortality in critically ill patients with acute pancreatitis (AP) in the intensive care unit, due to limited existing research on this correlation.

**Methods:**

This retrospective cohort study utilized data from the MIMIC-IV v2.0 and MIMIC-III v1.4 databases to investigate the potential relationship between NRBC levels and patient outcomes. The study employed restricted cubic splines (RCS) regression analysis to explore non-linear associations. The impact of NRBC on prognosis was assessed using a generalized linear model (GLM) with a logit link, adjusted for potential confounders. Furthermore, four machine learning models, including Gradient Boosting Classifier (GBC), Random Forest, Gaussian Naive Bayes, and Decision Tree Classifier model, were constructed using NRBC data to generate risk scores and evaluate the potential of NRBC in predicting patient prognosis.

**Results:**

A total of 354 patients were enrolled in the study, with 162 (45.8%) individuals aged 60 years or older and 204 (57.6%) males. RCS regression analysis demonstrated a non-linear relationship between NRBC levels and 90-day mortality. Receiver Operating Characteristic (ROC) analysis identified a 1.7% NRBC cutoff to distinguish survivor from non-survivor patients for 90-day mortality, yielding an Area Under the Curve (AUC) of 0.599, with a sensitivity of 0.475 and specificity of 0.711. Elevated NRBC levels were associated with increased risks of 90-day mortality in both unadjusted and adjusted models (all Odds Ratios > 1, *P* < 0.05). Assessment of various machine learning models with nine variables, including NRBC, Sex, Age, Simplified Acute Physiology Score II, Acute Physiology Score III, Congestive Heart Failure, Vasopressin, Norepinephrine, and Mean Arterial Pressure, indicated that the GBC model displayed the highest predictive accuracy for 90-day mortality, with an AUC of 0.982 (95% CI 0.970–0.994). Post hoc power analysis showed a statistical power of 0.880 in the study.

**Conclusions:**

Elevated levels of NRBC are linked to an increased mortality risk in critically ill patients with AP, suggesting its potential for predicting mortality.

**Supplementary Information:**

The online version contains supplementary material available at 10.1186/s12876-024-03444-z.

## Introduction

Acute pancreatitis (AP) is a prevalent gastrointestinal disorder characterized by a spectrum of severity, ranging from a mild form affecting solely the pancreas to a severe condition involving multisystem organ failure and mortality [1, 2]. Over the period from 1961 to 2016, the global incidence of AP demonstrated a 3.07% increase [3]. While most patients with AP exhibit mild interstitial edematous pancreatitis and achieve full recovery with supportive therapy within a few days, a subset exceeding 10% experience a more severe disease course necessitating intensive care unit (ICU) hospitalization [4]. Identifying risk factors associated with disease prognosis is essential for physicians to better anticipate disease evolution and patient outcomes, enhancing the provision of tailored treatment and care protocols.

Nucleated red blood cell (NRBC) represent immature forms of erythrocyte precursors that undergo a developmental process involving nucleus expulsion to become reticulocytes, which are the precursor cells to mature red blood cells [5, 6]. The rapid clearance of NRBC by the spleen underscores the efficient removal process within the peripheral blood, with the identification of circulating NRBC in adults often indicative of heightened erythropoietic activity or possible deficiencies in the filtration mechanisms of blood [7]. Such occurrences often indicate bone marrow damage or stress stimulation, which could suggest underlying serious health conditions [5, 8]. Numerous research studies have corroborated the effectiveness of NRBC as a biomarker for diagnosis and prognostication in critical care adult patients. In a prospective study involving medically intensive care patients, the presence of NRBC was identified in 17.5% of cases, with elevated NRBC levels associated with a significant rise in in-hospital mortality (50.7% vs. 9.8%) [9]. In an observational study conducted by Menk et al. [10], NRBC were found to be a predictive marker for mortality in acute respiratory distress syndrome. The study demonstrated an Area Under the Receiver Operating Characteristic (ROC) Curve of 0.71 (95% CI: 0.66–0.75), indicating the significance of NRBC as a prognostic indicator with statistical significance (*P* < 0.001) [10]. Furthermore, NRBC were associated with increased mortality in patients with surgical sepsis (27% vs. 12%; *P* < 0.001) [11].

There is a scarcity of investigations regarding the relationship between NRBC levels and mortality in critically ill AP patients. This study aimed to evaluate the influence of NRBC levels on mortality in critically ill AP patients. The study hypothesized that an escalation in NRBC levels would correlate with an increase in mortality among critically ill patients with AP.

## Methods

### Study design

The retrospective cohort study drew data from the MIMIC database. The study design procedures are depicted in Supplementary Fig. 1 to offer a comprehensive visual representation.

### Data sources

In line with the preceding statements, the data utilized in this study were sourced from the MIMIC databases, specifically the MIMIC-IV v2.0 and MIMIC-III v1.4 databases. These databases encompass comprehensive records of all intensive care patients admitted to the ICU at Beth Israel Deaconess Medical Center, providing a rich and varied patient dataset for our analysis (14). Data extraction and analysis were performed by RUAN (15), an author who has successfully completed the Collaborative Institutional Training Initiative (CITI) online training course and obtained authorization to collect and use data from the MIMIC databases (project approval number: 10520411).

### Sample size and power analysis

Given the retrospective design of the study, the sample size was predetermined [12]. Post-hoc power analyses were performed using PASS 15.0 software to assess the study’s statistical power. The calculated power from the analysis resulted in 0.880.

### Study population, inclusion, and exclusion criteria

The study focused on adult patients undergoing their initial admission to the ICU at Beth Israel Deaconess Medical Center, specifically diagnosed with AP. Inclusion criteria for the AP group were established according to the International Classification of Diseases (ICD) criteria conforming to both ICD-9 and ICD-10 classifications, as detailed in Supplementary Table 1. The exclusion criteria comprised of repeat admissions, pediatric cases, and patients with missing NRBC records. As depicted in Supplement Fig. 1, the combination of the MIMIC-IV v2.0 and MIMIC-III v1.4 databases initially encompassed 1,916 critically ill patients diagnosed with AP. After excluding minors (*n* = 2), and patients with missing NRBC recordings (*n* = 1560), the final study cohort comprised 354 patients.

### Grouping and study variables

In this study, the levels of NRBC were assessed within 24 h of the patient’s admission to the ICU. Patient demographic information included gender, age, race, specific treatments, medications, and existing medical conditions. Disease severity assessments were conducted within the first 24 h of admission to the ICU using an array of scoring systems to provide a comprehensive evaluation. These systems encompassed the Sequential Organ Failure Assessment (SOFA) score, which evaluates organ dysfunction, the Glasgow Coma Scale (GCS) for neurological assessment, the Simplified Acute Physiology Score II (SAPS II) for physiological stability, the Oxford Acute Severity of Illness Score (OASIS), the Logistic Organ Dysfunction System (LODS), and the Acute Physiology Score III (APS III) for multifaceted severity assessment [13–17]. The Glasgow Coma Scale (GCS) score was utilized as a standardized tool to evaluate and monitor the patient’s level of consciousness and neurological status [18, 19].

The categorical variables were stratified as follows: sex was categorized as male and female based on genetic sex, race was divided into white or non-white, age was segmented into elderly and non-elderly groups with a threshold age of 60 years, comorbidity was categorized based on the presence or absence of comorbidities, and drug use and non-drug use groups were determined by drug consumption or specialized treatments. The coding of comorbidities follows the definitions provided in the ICD9 and ICD10 codes [20]. Variance inflation factors were utilized to assess the covariance among the variables. The results of the multicollinearity tests are shown in Supplementary Table 2, indicating that all variance inflation factors (VIF) were below 10, suggesting no significant issues with multicollinearity.

### Clinical outcome

Approximately half of the fatalities associated with AP occur during the first 14 days, marking a pivotal period in the disease progression [21]. Furthermore, for late deaths, the median time to mortality extends to 56 days, with a range spanning from 19 to 81 days [21]. Consequently, the primary clinical endpoint addressed in this study centered on determining the 90-day all-cause mortality rate among critically ill individuals diagnosed with AP. Secondary clinical outcomes encompassed assessing the rates of 28-day all-cause mortality and in-ICU mortality to provide a comprehensive understanding of the disease course and patient outcomes.

### Data cleaning

The presence of missing data is a common challenge in clinical medicine datasets reflecting real-world conditions. In this dataset, only one missing value was detected, specifically in the GCS variable, with a count of *n* = 1 (Supplementary Fig. 2). This missing value was imputed using the median. Subsequently, an examination was conducted to assess the distribution of outliers for the continuous variables included in the dataset. Outliers were identified as data points exceeding 1.5 times the interquartile range (IQR) beyond the upper quartile (Q3) or falling below 1.5 times the IQR beneath the lower quartile (Q1) [22]. Referring to established methods for managing extreme outliers, the maximum value (Q3 + 1.5*IQR) or minimum value (Q1–1.5*IQR) was utilized to replace outliers (Supplementary Fig. 3) [23]. Moreover, to ensure the integrity and credibility of the study findings, supplementary sensitivity analyses were conducted utilizing the raw data. These additional analyses aimed to validate the robustness of the results and enhance the overall strength of the research outcomes.

### Tests for non-linear associations

To investigate the nonlinear relationship between NRBC levels and mortality, restricted cubic spline regression (RCS) was applied, with nodes positioned at the 5th, 35th, 65th, and 95th percentile values [24]. Additionally, a trend analysis was conducted by categorizing NRBC levels into three subsets based on trichotomies, which were included as dummy variables in the regression model.

### Constructing an NRBC-based mortality risk score

Creating a mortality risk assessment tool based on NRBC levels involved analyzing various factors using advanced statistical techniques like LASSO regression to support machine learning. Our study utilized four different machine learning models - Gaussian Naive Bayes (GNB), Decision Tree Classifier (DTC), Random Forest (RF), and Gradient Boosting Classifier (GBC) - to develop a predictive model based on NRBC levels for predicting mortality risk in patients with AP [25]. The individuals were randomly allocated into training and validation sets at a ratio of 7:3. The training set was employed to instruct the algorithm, the validation set facilitated model selection, while the test set evaluated the ultimate chosen model. The study assessed the effectiveness of each model using evaluation metrics such as the area under the ROC curve and the precision-recall (PR) curve. To understand better how NRBC levels influence outcomes in critically ill AP patients, the study applied the SHapley additive interpretation (SHAP) theory.

The machine learning algorithm with superior predictive capabilities was employed to calculate the probability of 90-day mortality for each patient. Following this, individuals were classified into high-risk and low-to-moderate risk categories using a tertile division based on their respective mortality probabilities. By comparing 90-day mortality between these risk categories, as depicted in Kaplan-Meier survival curves, we were able to assess the predictive accuracy of our model and identify potential differences in outcomes.

### Statistical analysis

Statistical analyses were performed using Stata 17.0 software (StataCorp, based in Texas, USA) and R software (version 4.3.0; developed by R Core Team in Vienna, Austria). Descriptive statistics for continuous variables were presented as either mean with standard deviation (SD) for normally distributed data or median with interquartile range (Q1 - Q3) for data that did not follow a normal distribution [26]. Normality of continuous data distribution in the dataset was assessed visually, as shown in Supplementary Fig. 4. Categorical variables were described as counts (n) and percentages (%) [27]. To compare non-normally distributed data sets, the Wilcoxon rank sum test was applied, while the Kruskal-Wallis test was used for comparisons involving multiple groups [28]. The chi-square test was utilized to evaluate associations between categorical variables.

Subgroup analyses and tests for multiplicative interactions were conducted for gender, race, comorbidity, treatment, and age variables. The influence of NRBC levels on patient prognosis was assessed through a generalized linear model (GLM) with a logit link, adjusting for potential confounders. This analysis aimed to investigate the relationship between NRBC levels and the outcomes of 90-day mortality, 28-day mortality, and in-ICU mortality. Statistical significance was defined as a two-tailed *P*-value of less than 0.05.

## Results

### Baseline characteristics of study population

In this study, a total of 354 critically ill patients with AP were included. The study cohort was stratified into survivor and non-survivor subsets based on the patients’ survival at 90 days post-admission. Table 1 shows a significant difference in NRBC levels between the non-survivor and survivor groups, with median values of 1 (1, 2) and 1 (1, 3), respectively (*P* < 0.001). In addition to these findings, the analysis revealed no significant variances in gender and age distributions between the two cohorts, further emphasizing the distinct NRBC levels as a potential marker for prognostic assessment. However, significant variances were evident in the age distribution, disease severity scores, utilization of vasoactive drugs, and the presence of concurrent heart failure and renal failure between the survivor and non-survivor groups (all *P* < 0.05).

**Table 1:** Baseline information of patients included in this study

Characteristics	Overall (*n* = 354)	Survivor (*n* = 232)	Non-survivor (*n* = 122)	*P* value
NRBC, %	1 (1, 2)	1 (1, 2)	1 (1, 3)	**< 0.001**
Sex, n (%)				0.330
Female	150 (42.4%)	94 (40.5%)	56 (45.9%)	
Male	204 (57.6%)	138 (59.5%)	66 (54.1%)	
Race, n (%)				0.857
White	217 (61.3%)	143 (61.6%)	74 (60.7%)	
Non-white	137 (38.7%)	89 (38.4%)	48 (39.3%)	
Age, n (%)	57 (46, 70)	52 (42, 65)	67 (52, 78)	**< 0.001**
<60	192 (54.2%)	146 (62.9%)	46 (37.7%)	
≥60	162 (45.8%)	86 (37.1%)	76 (62.3%)	
MAP, mmHg	77 (56, 100)	79 (58, 104)	71 (52, 95)	**0.016**
Critical Care Score				
SOFA	9 (5, 12)	8 (5, 11)	10 (7, 13)	**< 0.001**
SAPSII	44 (33, 55)	40 (28.75, 53.25)	50 (41, 58.75)	**< 0.001**
OASIS	39 (31, 46)	37 (30, 44)	42 (34, 48)	**< 0.001**
LODS	7 (4.25, 10)	6 (4, 9)	8 (6, 11)	**< 0.001**
APSIII	68.5 (49, 90)	62 (44, 84)	78 (63, 101.75)	**< 0.001**
GCS	9 (3, 14)	14 (9, 15)	13 (9, 15)	0.417
Co-morbidity				
Diabetes, n (%)	100 (28.2%)	64 (27.6%)	36 (29.5%)	0.703
CHF, n (%)	93 (26.3%)	49 (21.1%)	44 (36.1%)	**0.002**
CPD, n (%)	69 (19.5%)	42 (18.1%)	27 (22.1%)	0.363
Renal disease, n (%)	69 (19.5%)	36 (15.5%)	33 (27.0%)	**0.009**
Cerebrovascular disease, n (%)	30 (8.5%)	22 (9.5%)	8 (6.6%)	0.348
Treatment				
Vasopressin, n (%)	116 (32.8%)	54 (23.3%)	62 (50.8%)	**< 0.001**
Norepinephrine, n (%)	183 (51.7%)	100 (43.1%)	83 (68%)	**< 0.001**
RRT, n (%)	29 (8.2%)	17 (7.3%)	12 (9.8%)	0.413

Abbreviations: APS III, Acute Physiology Score III; CHF, Congestive Heart Failure; CPD, Chronic Pulmonary Disease; GCS, Glasgow Coma Scale; LODS, Logistic Organ Dysfunction System; MAP, Mean Arterial Pressure; NRBC, Nucleated Red blood Cells; OASIS, Oxford Acute Severity of Illness Score; RRT, renal replacement therapy; SAPS II, Simplified Acute Physiology Score II; SOFA, Sequential Organ Failure Assessment

### NRBC exhibited a correlation with the severity of the disease

**Fig. 1:**
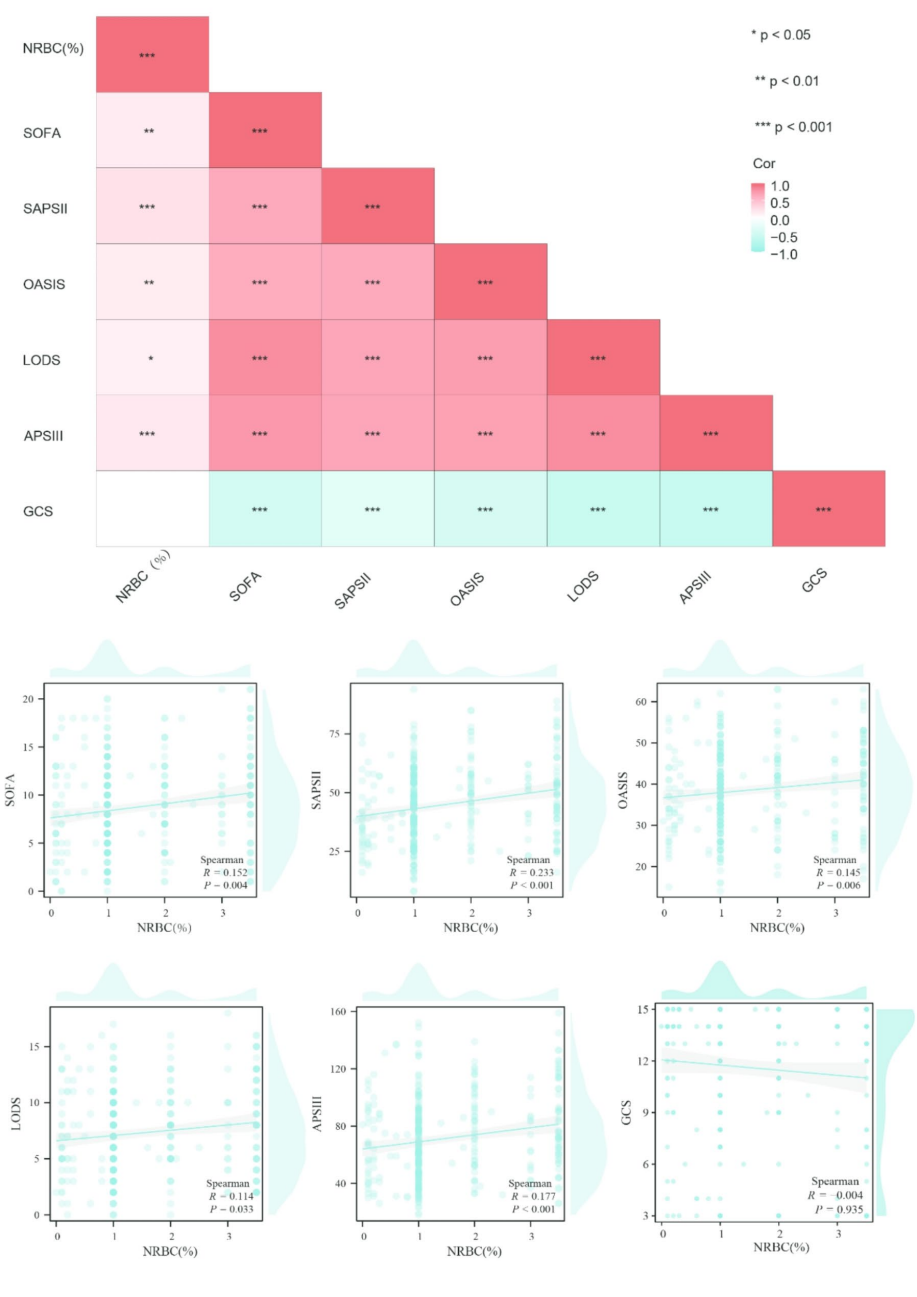
Correlation analysis between NRBC and disease scores

Spearman’s correlation analysis identified significant but weak positive correlations between NRBC levels and various severity scores, except for the GCS score (all Spearman’s |*R*| < 0.3, Fig. 1). The strongest correlation was observed between NRBC levels and SAPS II (*R* = 0.233, *P* < 0.001), followed by APS III score (*R* = 0.177, *P* < 0.001), SOFA score (*R* = 0.152, *P* < 0.001), OASIS score (*R* = 0.145, *P* = 0.006), and LODS score (*R* = 0.114, *P* < 0.001). These findings suggest a potential association, indicating that higher NRBC levels may be linked to increased disease severity

### Non-linear association between NRBC and mortality

**Fig. 2:**
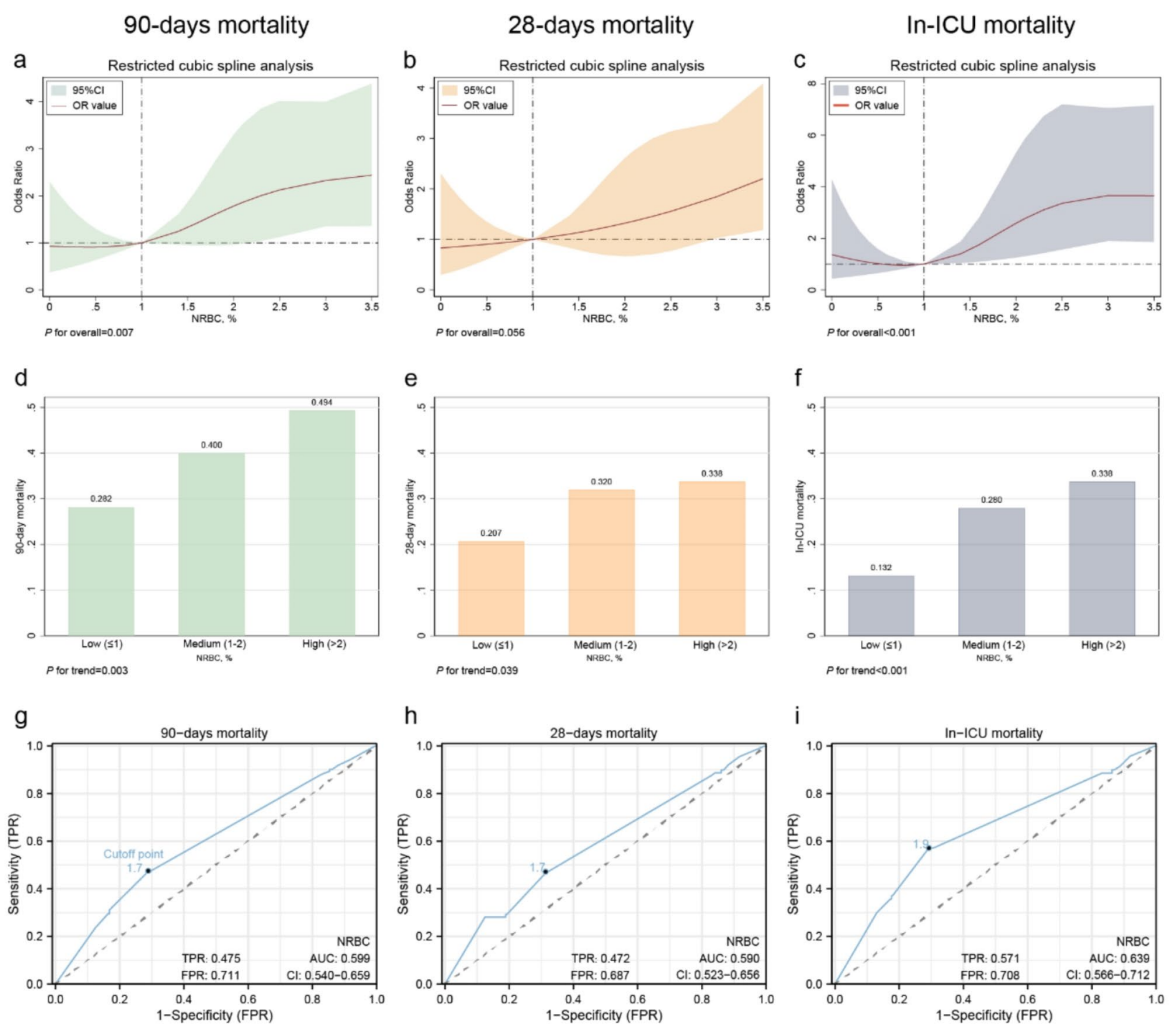
Association of NRBC with 90-Day Mortality, 28-Day Mortality, and ICU Mortality (**a-c**) RCS regression; (**d-f**) Grouped regression; (**g-i**) ROC curve. NOTE: The high, medium, and low groupings of NRBC in Fig. 2**d-f** are established according to the tertile divisions of NRBC levels

The RCS regression model identified a nonlinear relationship between NRBC levels and 90-day mortality, as well as in-ICU mortality (*P* for overall < 0.001, Fig. 2a, b). In contrast, no significant nonlinear association was observed between 28-day mortality and NRBC levels (*P* for overall > 0.05, Fig. 2c). Subsequently, NRBC levels were stratified into three categories using tertiles: low (NRBC ≤ 1%, *n* = 227), medium (1–2%, *n* = 50), and high (NRBC > 2%, *n* = 77). These groups exhibited increasing trends in all three mortality indicators with higher NRBC levels (90-day mortality: Low vs. Medium vs. High group = 28.2% vs. 40.0% vs. 49.4%; 28-day mortality: Low vs. Medium vs. High group = 20.7% vs. 32.0% vs. 33.8%; In-ICU mortality: Low vs. Medium vs. High group = 13.7% vs. 28.0% vs. 33.8%; all *P* for trend < 0.05; Fig. 2d-f). ROC curve analysis determined a cutoff NRBC value of 1.7% to differentiate between survivors and non-survivors for 90-day and 28-day mortality, while the cutoff for in-hospital mortality was an NRBC of 1.9% (Fig. 2g-i)

### Results of subgroup analyses

**Fig. 3:**
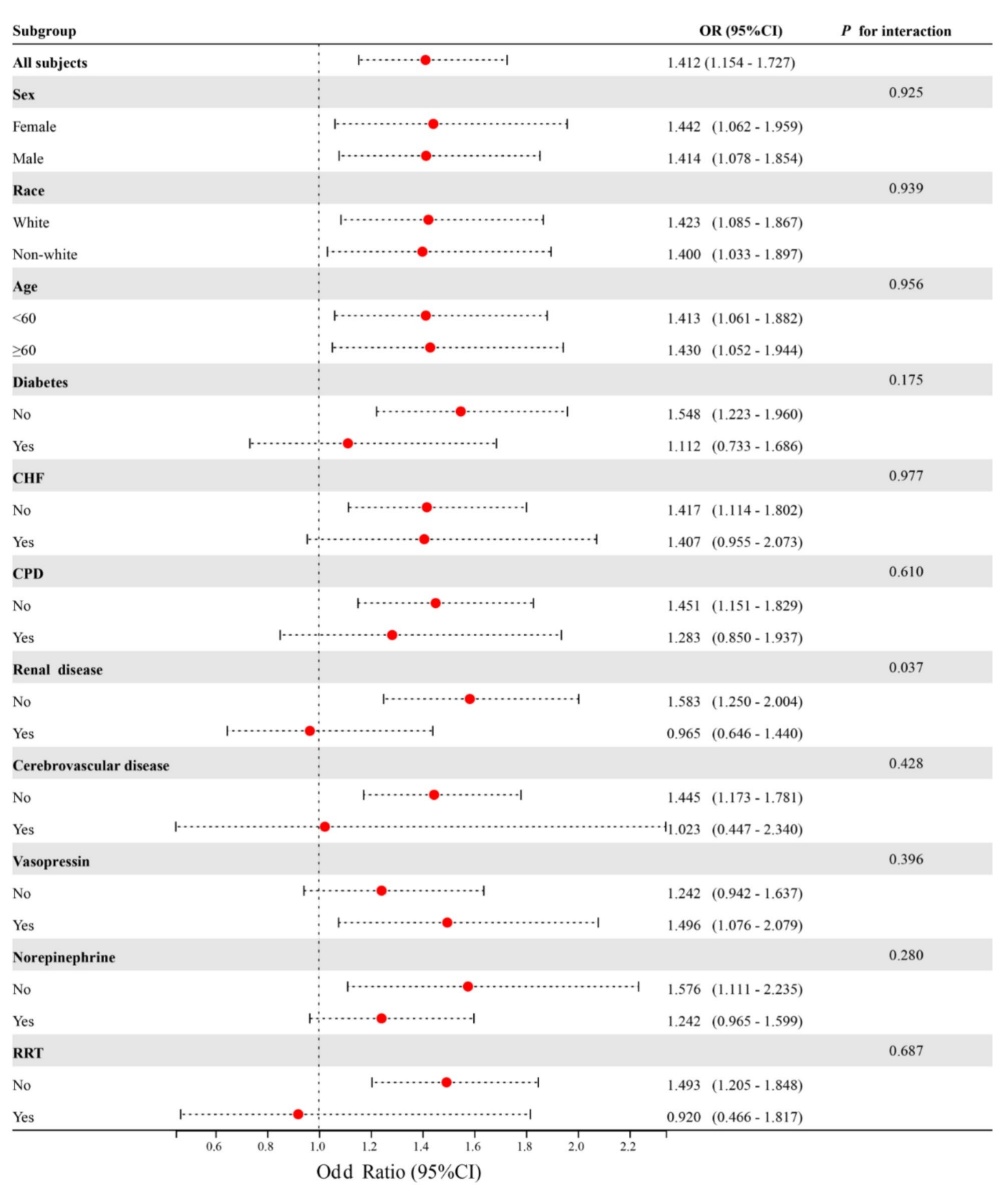
Effect size of NRBC on 90-day mortality in prespecified and exploratory subgroups

To assess potential clinical heterogeneity, this study utilized interaction and stratification analyses (Fig. 3). The findings revealed a significant interaction between NRBC and chronic kidney disease (*P* for interaction < 0.05). Notably, no significant interactions or stratified analyses were observed for variables such as age (< 60 and ≥ 60 years), sex, race, diabetes mellitus, chronic heart failure, cerebrovascular disease, use of angiotensin, norepinephrine, and Renal Replacement Therapy (RRT)

### NRBC as independent prognostic indicator for 90-day mortality

In this study, GLM-based univariate and multivariate analyses were conducted to assess the prognostic impact of NRBC. The univariate logistic regression analyses revealed a significant association between increased NRBC levels and 90-day mortality (Odds ratio (OR) (95% confidence intervals (CI)) = 1.412 (1.154–1.727), *P* < 0.001, Table 2), suggesting that heightened NRBC levels act as a risk factor for the occurrence of 90-day mortality in critically ill patients with AP. After a comprehensive multivariate analysis using a GLM regression and adjusting for key variables including age, comorbidities, and critical care scores, the results consistently highlighted the significant association of NRBC levels with 90-day mortality. Specifically, the adjusted OR and its corresponding 95% (CI)were calculated as 1.282 (1.020–1.611), with a statistically significant *P*-value of 0.033, as detailed in Table 2. These findings underscore the potential prognostic value of NRBC levels in critically ill AP patients.

**Table 2:** Univariate and Multivariate GLM Regression Analysis Investigating Association with 90-Day mortality

Characteristics	Univariate analysis			Multivariate analysis	
	Odds Ratio (95% CI)	*P*-value		Odds Ratio (95% CI)	*P*-value
NRBC, %	1.412 (1.154–1.727)	**< 0.001**		1.282 (1.020–1.611)	**0.033**
Sex (Female vs. Male)	0.803 (0.516–1.249)	0.330			
Race (While vs. Non-while)	1.042 (0.665–1.634)	0.857			
Age (< 60 vs. ≥60)	2.805 (1.783–4.411)	**< 0.001**		2.038 (1.172–3.541)	**0.012**
CHF (No vs. Yes)	2.107 (1.296–3.424)	**0.003**		1.743 (0.963–3.155)	0.066
CPD (No vs. Yes)	1.286 (0.747–2.212)	0.364			
Diabetes (No vs. Yes)	1.099 (0.677–1.783)	0.703			
Renal disease (No vs. Yes)	2.019 (1.183–3.446)	**0.010**		1.057 (0.561–1.993)	0.864
Cerebrovascular disease (No vs. Yes)	0.670 (0.289–1.553)	0.350			
Vasopressin (No vs. Yes)	3.406 (2.134–5.437)	**< 0.001**		1.792 (0.947–3.391)	0.073
Norepinephrine (No vs. Yes)	2.809 (1.772–4.453)	**< 0.001**		1.528 (0.778–3.001)	0.218
RRT (No vs. Yes)	1.380 (0.636–2.991)	0.415			
SOFA	1.096 (1.044–1.150)	**< 0.001**		0.983 (0.887–1.089)	0.739
SAPSII	1.044 (1.028–1.061)	**< 0.001**		1.027 (1.001–1.053)	**0.043**
OASIS	1.042 (1.019–1.067)	**< 0.001**		0.979 (0.941–1.018)	0.280
LODS	1.129 (1.064–1.199)	**< 0.001**		0.968 (0.844–1.110)	0.640
APSIII	1.019 (1.011–1.027)	**< 0.001**		1.011 (0.993–1.028)	0.229
GCS	0.980 (0.930–1.032)	0.442			
MAP, mmHg	0.991 (0.984–0.998)	**0.015**		0.994 (0.987–1.002)	0.160

Abbreviations: APS III, Acute Physiology Score III; CHF, Congestive Heart Failure; CPD, Chronic Pulmonary Disease; GCS, Glasgow Coma Scale; GLM, generalized linear model; LODS, Logistic Organ Dysfunction System; MAP, Mean Arterial Pressure; NRBC, Nucleated Red blood Cells; OASIS, Oxford Acute Severity of Illness Score; RRT, renal replacement therapy; SAPS II, Simplified Acute Physiology Score II; SOFA, Sequential Organ Failure Assessment

### NRBC levels as risk factor for mortality

This study further examined the influence of NRBC levels on 90-day mortality, 28-day mortality, and in-ICU mortality using different models (Table 3). In the unadjusted original model, NRBC level emerged as a significant risk factor for all mortality outcomes (90-day mortality: OR (95% CI) = 1.412 (1.154–1.727); 28-day mortality: OR (95% CI) = 1.349 (1.088–1.673); in-ICU mortality: OR (95% CI) = 1.586 (1.258–2.000); all *P* < 0.01). Model 1, adjusting for age and sex, indicated that NRBC levels remained a risk factor for all mortality endpoints (all OR > 1, *P* < 0.01). Similarly, in Model 2, which further adjusted for age, sex, and race, NRBC levels were consistently associated with increased mortality risk (all OR > 1, *P* < 0.01). Model 3, encompassing all confounding factors affecting prognosis, confirmed that NRBC level continued to be a risk factor for both 90-day mortality and In-ICU mortality (90-day mortality: OR (95% CI) = 1.282 (1.020–1.611); In-ICU mortality: OR (95% CI) = 1.444 (1.084–1.923); *P* < 0.05). However, NRBC levels did not demonstrate a significant impact on 28-day mortality outcomes (OR (95% CI) = 1.162 (0.903–1.494), *P* = 0.242).

**Table 3:** Effect of NRBC on primary and secondary clinical outcomes

Model (All subjects = 354)	90-day mortality (*n* = 122, 34.46%)	28-day mortality (*n* = 89, 25.14%)	In-ICU mortality (*n* = 70, 19.77%)
Crude model OR (95% CI)	1.412 (1.154–1.727)	1.349 (1.088–1.673)	1.586 (1.258–2.000)
*P*-value	**< 0.001**	**0.006**	**< 0.001**
Adjusted Model1 OR (95% CI)	1.438 (1.165–1.776)	1.391 (1.108–1.746)	1.627 (1.284–2.062)
*P*-value	**0.001**	**0.004**	**< 0.001**
Adjusted Model2 OR (95% CI)	1.436 (1.162–1.776)	1.374 (1.093–1.726)	1.609 (1.269–2.041)
*P*-value	**0.001**	**0.006**	**< 0.001**
Adjusted Model3 OR (95% CI)	1.282 (1.020–1.611)	1.162 (0.903–1.494)	1.444 (1.084–1.923)
*P*-value	**0.033**	0.242	**0.012**

Note: Crude model: unadjusted for confounding factors; Model1: adjusted for sex and age; Model2: adjusted for sex, race, and age; Model3: adjusted for age, CHF, Renal disease, Vasopressin, Norepinephrine, SOFA, SAPSII, OASIS, LODS, APSIII, and MAP. Abbreviations: APS III, Acute Physiology Score III; CHF, Congestive Heart Failure; LODS, Logistic Organ Dysfunction System; MAP, Mean Arterial Pressure; NRBC, Nucleated Red blood Cells; OASIS, Oxford Acute Severity of Illness Score; SAPS II, Simplified Acute Physiology Score II; SOFA, Sequential Organ Failure Assessment

### The NRBC-based risk score efficiently predicts 90-day mortality

**Fig. 4:**
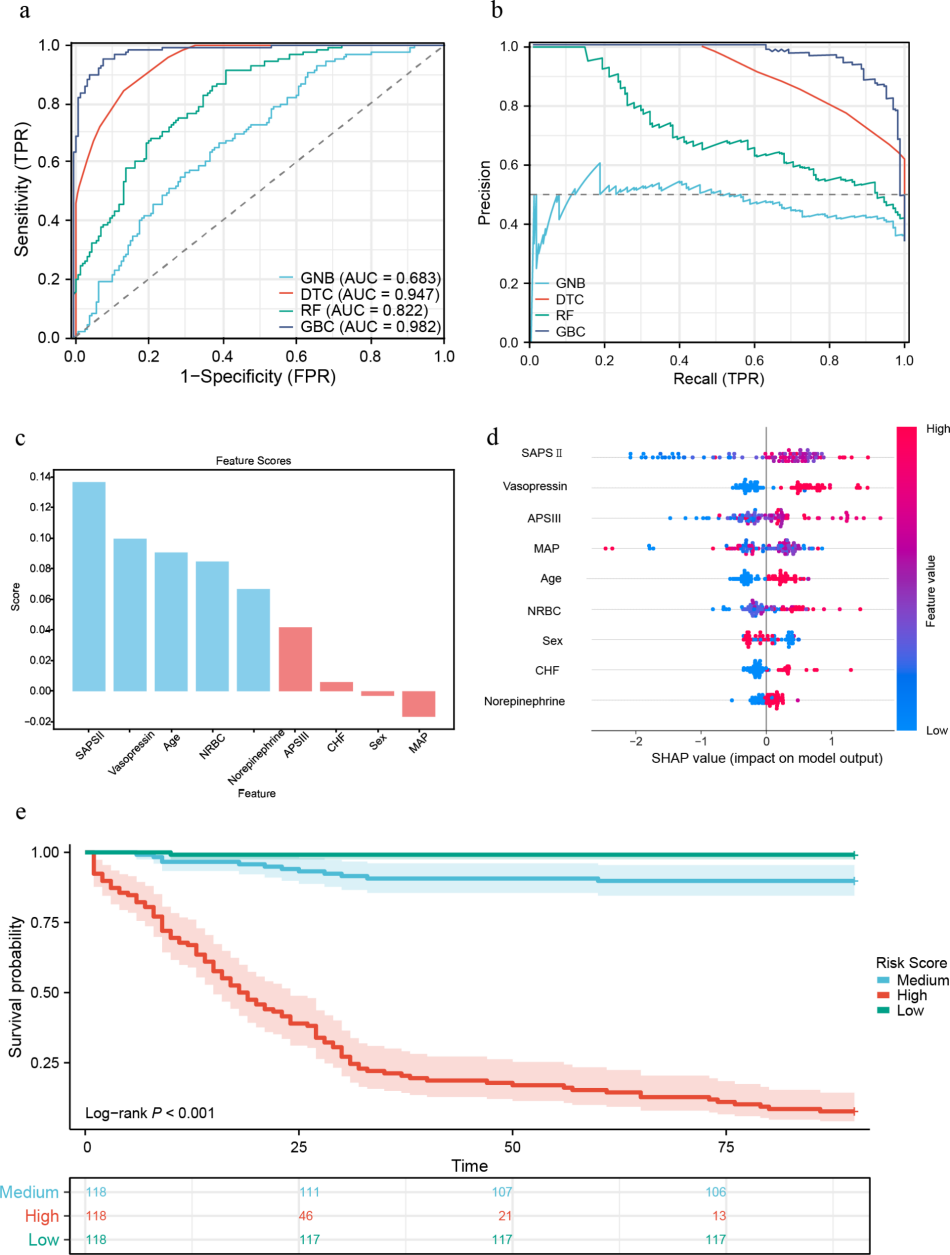
The NRBC-based risk score efficiently predicts 90-day Mortality (**a**) ROC curves for the performance of various machine learning models in predicting 90-day mortality are as follows: GBC model AUC (95% CI) = 0.982 (0.970–0.994); DTC model AUC (95% CI) = 0.947 (0.928–0.967); RF model AUC (95% CI) = 0.822 (0.779–0.865); GNB model AUC (95% CI) = 0.683 (0.627–0.739); (**b**) Precision-Recall curves for various machine learning models illustrate performance metrics, with a greater curve towards the upper right corner indicating superior model performance.; (**c**) Ranking of feature variable importance in the GBC models; (**d**) SHapley Additive Explanation visualization illustrating the individual feature impacts on the outcomes of the GBC prediction model. The color spectrum represents the degree of influence, where redder hues indicate a higher association with the risk of 90-day Mortality, while bluer hues suggest a lower association with the risk of 90-day Mortality; (**e**) Survival curve analysis in subgroups with different risk scores. NOTE: The three subgroups for the survival curve analyses were established based on the tertiles derived from the mortality probability generated by the GBC prognostic model constructed using the 9 variables

Through LASSO regression, nine variables, including NRBC, Sex, Age, SAPSII, APSIII, CHF, Vasopressin, Norepinephrine, and MAP, were evaluated for inclusion in the machine learning models (Supplementary Fig. 5a, b). Subsequently, four distinct machine learning models were generated based on the features selected by LASSO regression, with the parameter optimization process demonstrated in Supplementary Fig. 5c-f. Among the models, the GBC model demonstrated the most effective predictive performance, highlighted by both the ROC curve and diagnostic PR curve (AUC (95% CI) = 0.982(0.970–0.994), Fig. 4a, b). Notably, NRBC ranked fourth in terms of feature importance within the model (Feature NRBC score: 0.085, Fig. 4c). Further interpretation from SHAP analysis revealed a direct correlation between elevated levels of NRBC and an increased risk of mortality in patients with AP (Fig. 4d). Based on the risk scores classified as high, medium, and low, survival curves illustrated notable discrepancies among the different risk subgroups (log-rank *P* < 0.001, Fig. 4e).

### Sensitivity analysis

To ensure the robustness of the findings, this study conducted two sensitivity analyses. Firstly, the raw data were reanalyzed, and consistent results were observed across all scenarios (Supplementary Tables 3–4, Supplementary Figs. 6–7). Secondly, NRBC levels were categorized as a discrete variable, dividing participants into three subgroups representing low, intermediate, and high NRBC levels. Kaplan-Meier survival analyses depicted that high NRBC levels were associated with poorer outcomes compared to low NRBC levels (log-rank test, *P* = 0.001, Supplementary Fig. 8).

## Discussion

The primary aim of this study was to explore the correlation between NRBC levels and mortality in patients with AP. Our findings indicated significantly elevated NRBC levels in non-surviving AP patients compared to survivors. Both univariate and multivariate GLM regression analyses recognized NRBC as an independent predictor of 90-day mortality in this patient cohort. NRBC levels exhibited a consistent association with 90-day mortality across various confounder-corrected models, underscoring the robustness of the results. Nonlinear correlation analysis unveiled a non-linear impact of NRBC levels on 90-day mortality. Moreover, the study established the moderate predictive value of NRBC levels for 90-day mortality in AP patients, identifying a cut-off point of 1.7% through ROC curve analysis. Additionally, four machine learning models based on NRBC levels were developed and evaluated, with the optimal prognostic model determined and its predictive performance assessed in patients with AP. Overall, the study highlighted the practical clinical utility of NRBC in prognosticating outcomes in patients with AP.

This finding is supported by prior clinical studies highlighting the importance of NRBC as a significant clinical predictor of mortality in critically ill patients [5, 9, 29–31]. In healthy individuals, NRBC are typically absent in peripheral blood; however, their presence in acute care settings often signifies severely ill patients with a less favorable prognosis [29]. A retrospective study by Shah et al. [29], involving 9,690 patients in a surgical ICU, identified a correlation between elevated NRBC levels and heightened mortality risk among critically ill surgical patients. Similarly, a prospective cohort study in a cardiac ICU, focusing on patients with cardiovascular disease, demonstrated that NRBC could serve as a prognostic indicator for all-cause mortality in individuals with cardiovascular issues [30]. This association was also observed among COVID-19 patients admitted to the ICU [31]. Additionally, in agreement with Stachon et al. [32], our study found that NRBC did not significantly impact individuals with concurrent chronic kidney disease, suggesting that the presence of NRBC in the blood may not be closely related to renal failure.

The study by Xu et al. [33] conducted a retrospective analysis involving 92 severe pancreatitis patients to develop a mortality prediction model for severe acute pancreatitis (SAP) by incorporating NRBC levels. Their research highlighted that NRBC, when integrated with factors such as CCIs, APACHE II, and Ranson score, could serve as indicators for predicting 90-day mortality in SAP patients. Nevertheless, the limited number of positive outcome events (*n* = 11) in their study might constrain the applicability of their model. Additionally, their study was confined to general ward patients and did not encompass data from ICU patients. Building upon the insights from this prior research, our study delves into a more comprehensive assessment of NRBC levels in critically ill AP patients. In the study, the optimal cut-off point for NRBC levels was determined to be 1.7% based on the ROC curve analysis for predicting 90-day mortality, with a sensitivity of 0.475 and a specificity of 0.711. While these values suggest medium-low sensitivity and specificity in predicting severity, a machine learning strategy was employed in our approach to develop a prognostic model that integrated NRBC levels with other clinical parameters, effectively enhancing the predictive performance of the model (GBC model AUC = 0.982). This illustrates the practical application of utilizing NRBC levels in clinical medicine.

Elevated levels of NRBC in the intensive care setting may signify poor outcomes, but the exact mechanisms by which NRBC contributes to increased mortality in critically ill patients are not yet fully understood. Various studies have provided potential insights from different perspectives. Firstly, hypoxemia is more prevalent in NRBC-positive patients compared to NRBC-negative individuals [34]. Secondly, rapid hemolysis or acute blood loss can result in NRBC presence in the bloodstream, possibly due to enhanced erythropoiesis as a compensatory response to acute anemia [6]. Lastly, infections may also contribute to elevated NRBC levels [35]. NRBC-positive patients tend to have higher levels of cytokines and erythropoietin compared to NRBC-negative patients, suggesting that NRBC can potentially serve as biomarkers for hypoxia and inflammatory damage [36]. Our study demonstrated that elevated NRBC levels are an independent prognostic indicator for 90-day mortality, supporting previous findings while expanding the potential applications of this research.

One of the strengths of this study is its large sample size sourced from a publicly available dataset, enabling a comprehensive exploration of the relationship between NRBC levels and prognosis in critically ill patients with AP. Unlike prior studies that primarily focus on patients in general wards, this study emphasizes patients with AP in ICU, contributing valuable insights to the literature concerning this specific patient population. The study utilized a robust study design and rigorous statistical methods to enhance the reliability of the findings, particularly by investigating the non-linear relationship between NRBC levels and clinical prognosis. However, the study has some limitations. Firstly, being retrospective, it could only establish an association between NRBC levels and 90-day mortality without proving causality and might have overlooked potential confounding variables influencing the results. Secondly, the absence of specific subtypes of AP in the database could have impacted the outcomes.

Overall, this study underscores the significance of NRBC as a prognostic marker for AP and underscores the importance of integrating NRBC assessment into the management of patients with this condition. Future research could expand on this work with larger prospective studies to further validate the prognostic value of NRBC in critically ill AP patients.

## Conclusions

The elevation of NRBC levels correlates with an increased risk of 90-day all-cause mortality in critically ill patients with AP, suggesting that NRBC has the potential to be incorporated with other indicators to establish a predictive model for mortality forecasting.

## Electronic supplementary material

Below is the link to the electronic supplementary material.

Supplementary Material 1

## Data Availability

The datasets and codes employed in this study can be accessed from the corresponding author upon reasonable request.

## Abbreviations

APS III: Acute Physiology Score III
AP: Acute pancreatitis
CHF: Congestive Heart Failure
CI: Confidence intervals
CITI: Collaborative Institutional Training Initiative
CPD: Chronic Pulmonary Disease
DTC: Decision Tree Classifier
GBC: Gradient Boosting Classifier
GCS: Glasgow Coma Scale
GLM: Generalized linear model
GNB: Gaussian Naive Bayes
ICD: International Classification of Diseases
ICU: Intensive care unit
IQR: Interquartile range
LODS: Logistic Organ Dysfunction System
MAP: Mean Arterial Pressure
NRBC: Nucleated Red Blood Cell
OASIS: Oxford Acute Severity of Illness Score
OR: Odds ratio
PR: Precision-recall
RBC: Restricted Cubic Spline
RF: Random Forest Classifier
ROC: Receiver operating characteristic
RRT: Renal Replacement Therapy
SAP: Severe Acute Pancreatitis
SAPS II: Simplified Acute Physiology Score II
SD: Standard Deviation
SHAP: SHapley Additive Interpretation
SOFA: Sequential Organ Failure Assessment
STROBE: Strengthening the Reporting of Observational studies in Epidemiology
