# Supplementary material for: Nucleated red blood cell distribution in critically ill patients with acute pancreatitis: a retrospective cohort study
Source: BMC Gastroenterol. 2024 Oct 7;24:353. doi: 10.1186/s12876-024-03444-z (PMC11460230; doi:10.1186/s12876-024-03444-z)
Supplement: Supplementary file 1 — Supplementary Material 1 [file 12876_2024_3444_MOESM1_ESM.docx]

**Nucleated red blood cell distribution in critically ill patients with acute pancreatitis: a retrospective cohort study**

Huan-qin Liu^1^; Guan-qun Wang^1^; Cheng-shuang Zhang^1^; Xia Wang^1^; Ji-kui Shi ^1#^; Feng Qu^1#^; Hang Ruan^2,3^

^1^ Department of Critical-care Medicine, Jining NO.1 People's Hospital, Jining, 272000, Shandong Province, China

^2^ Department of Critical-care Medicine, Tongji Hospital, Tongji Medical College, Huazhong University of Science and Technology, Wuhan, 430030, Hubei Province, China;

^3^ Department of Emergency Medicine, Tongji Hospital, Tongji Medical College, Huazhong University of Science and Technology, Wuhan, 430030, Hubei Province, China;

**^#^Corresponding authors 1:** Ji-kui Shi, E-mail: sjkjnrmyy@126.com, Tel: 0086- 15965721736

**^#^Corresponding authors 2:** Feng Qu, E-mail: rmyyzzeq@163.com, Tel: 0086- 18678769833

**Key words**: Critical care; Cohort study; Nucleated red blood cell; Acute Pancreatitis; Risk factors

**CONFLICT OF INTEREST**

The authors declare that they have no known competing financial interests or personal relationships that could have appeared to influence the work reported in this paper.

**FUNDING**

This work was supported by funding from the Shandong Province medical health science and technology project. (202319010449).

Content

[Supplementing Figure 1 4](#_Toc169681662)

[Supplementing Figure 2 5](#_Toc169681663)

[Supplementing Figure 3 6](#_Toc169681664)

[Supplementing Figure 4 7](#_Toc169681665)

[Supplementing Figure 5 8](#_Toc169681666)

[Supplementing Figure 6 9](#_Toc169681667)

[Supplementing Figure 7 10](#_Toc169681668)

[Supplementing Figure 8 11](#_Toc169681670)

[Supplementing Table 1: ICD Codes for Acute Pancreatitis 12](#_Toc169681671)

[Supplementing Table 2: Collinearity Test 13](#_Toc169681672)

[Supplementing Table 3: Univariate and multivariate analyses using raw data 14](#_Toc169681673)

[Supplementing Table 4: Effect size of NRBC on mortality using raw data 15](#_Toc169681674)

# Supplementing Figure 1


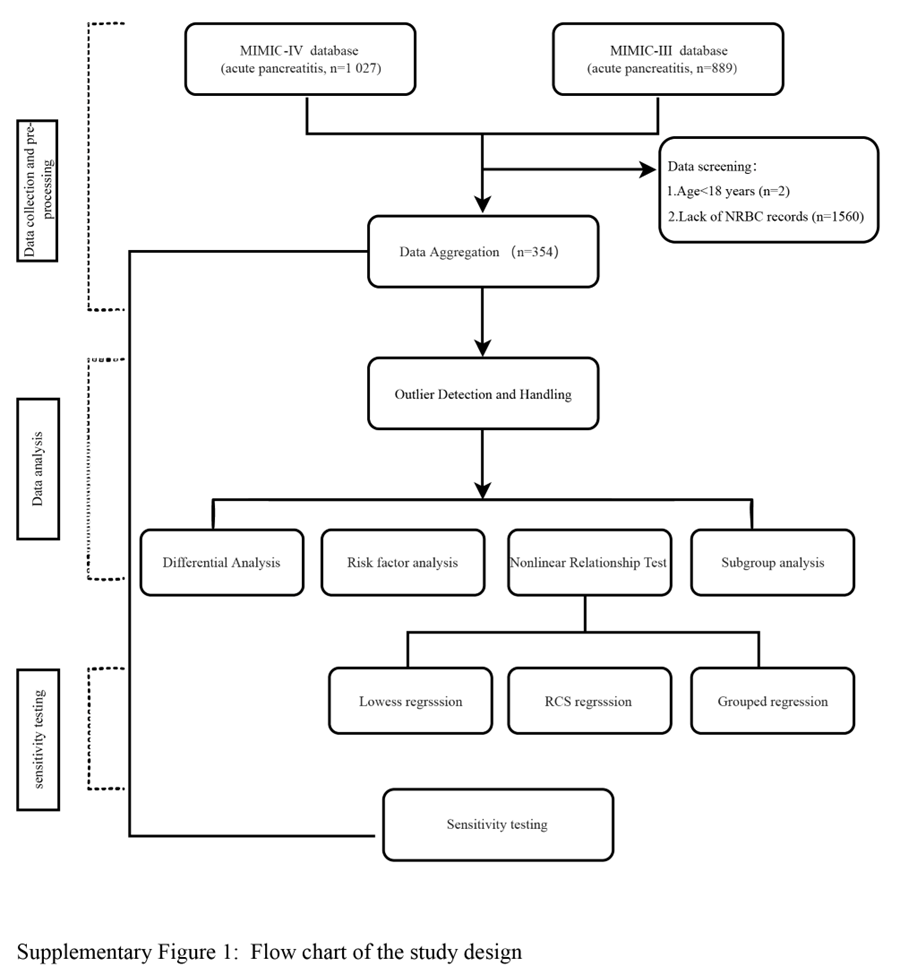


# Supplementing Figure 2


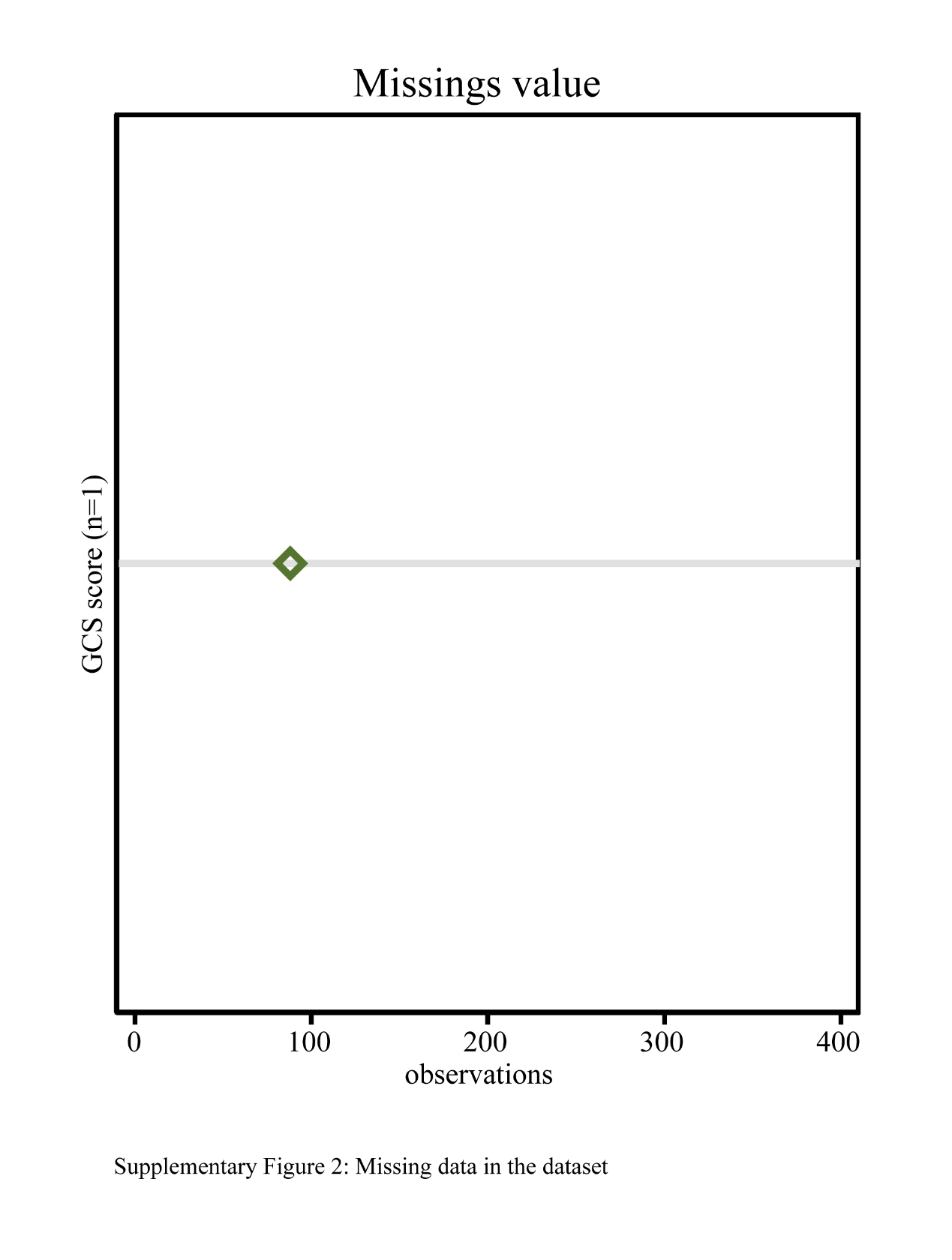


# Supplementing Figure 3


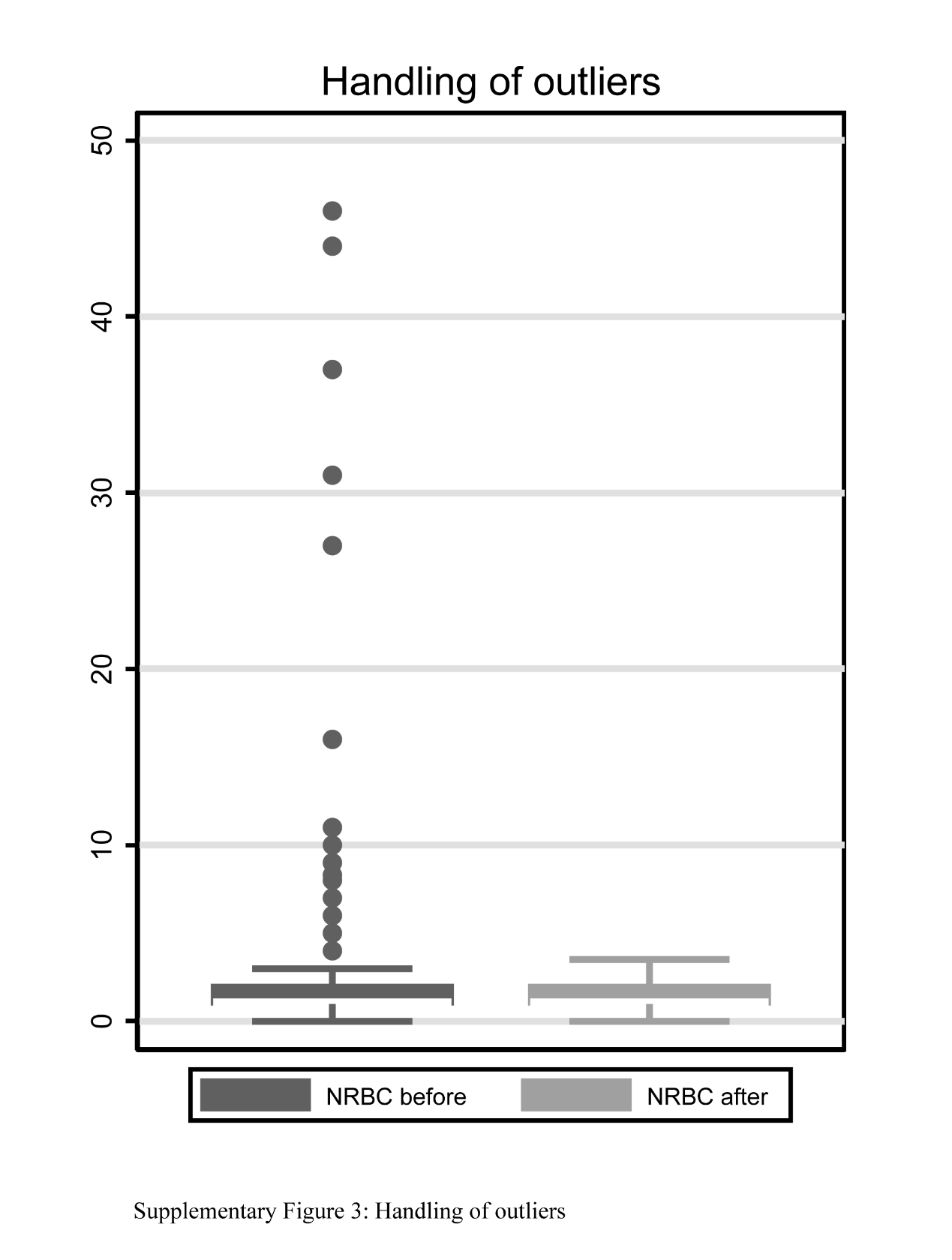


# Supplementing Figure 4


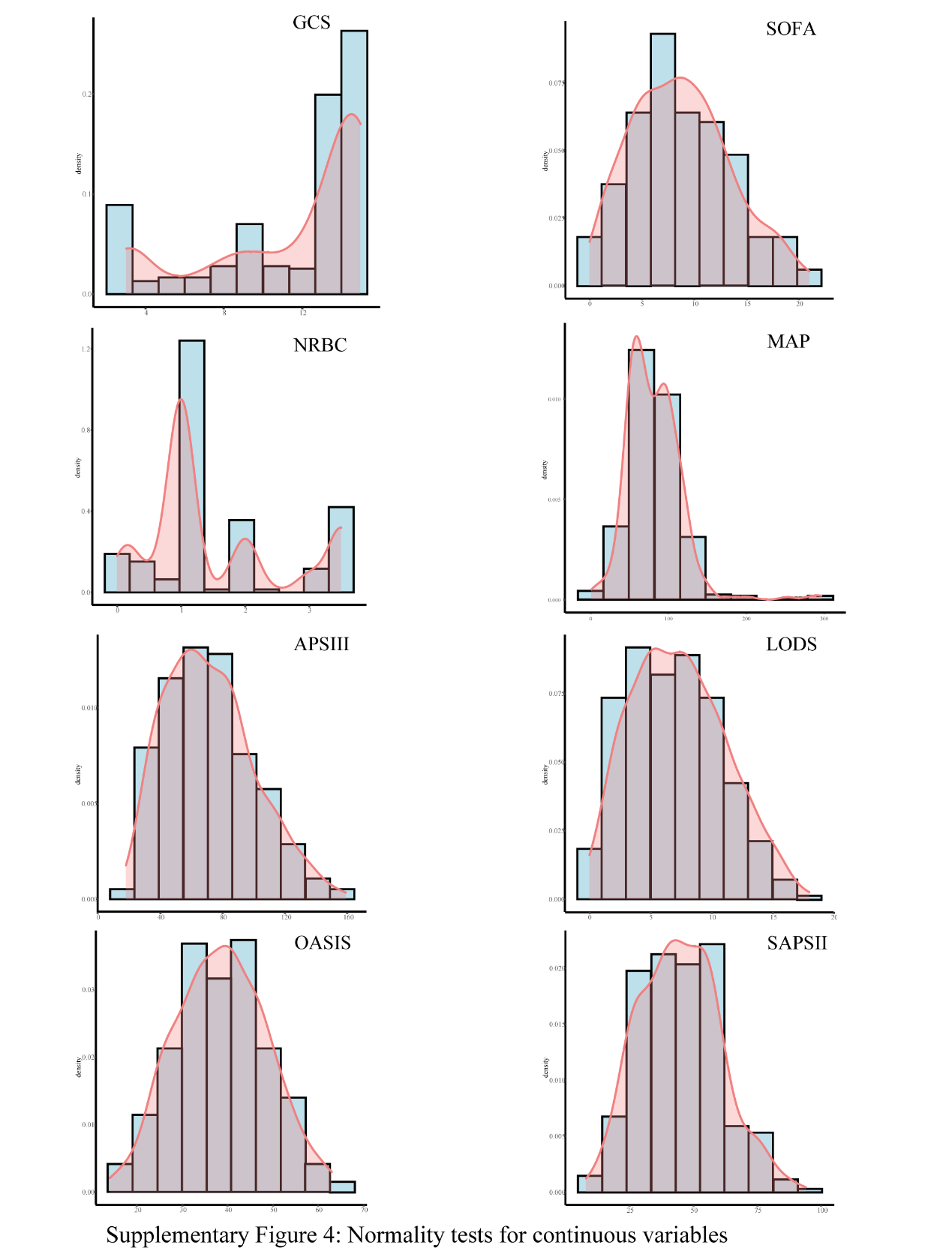


# Supplementing Figure 5


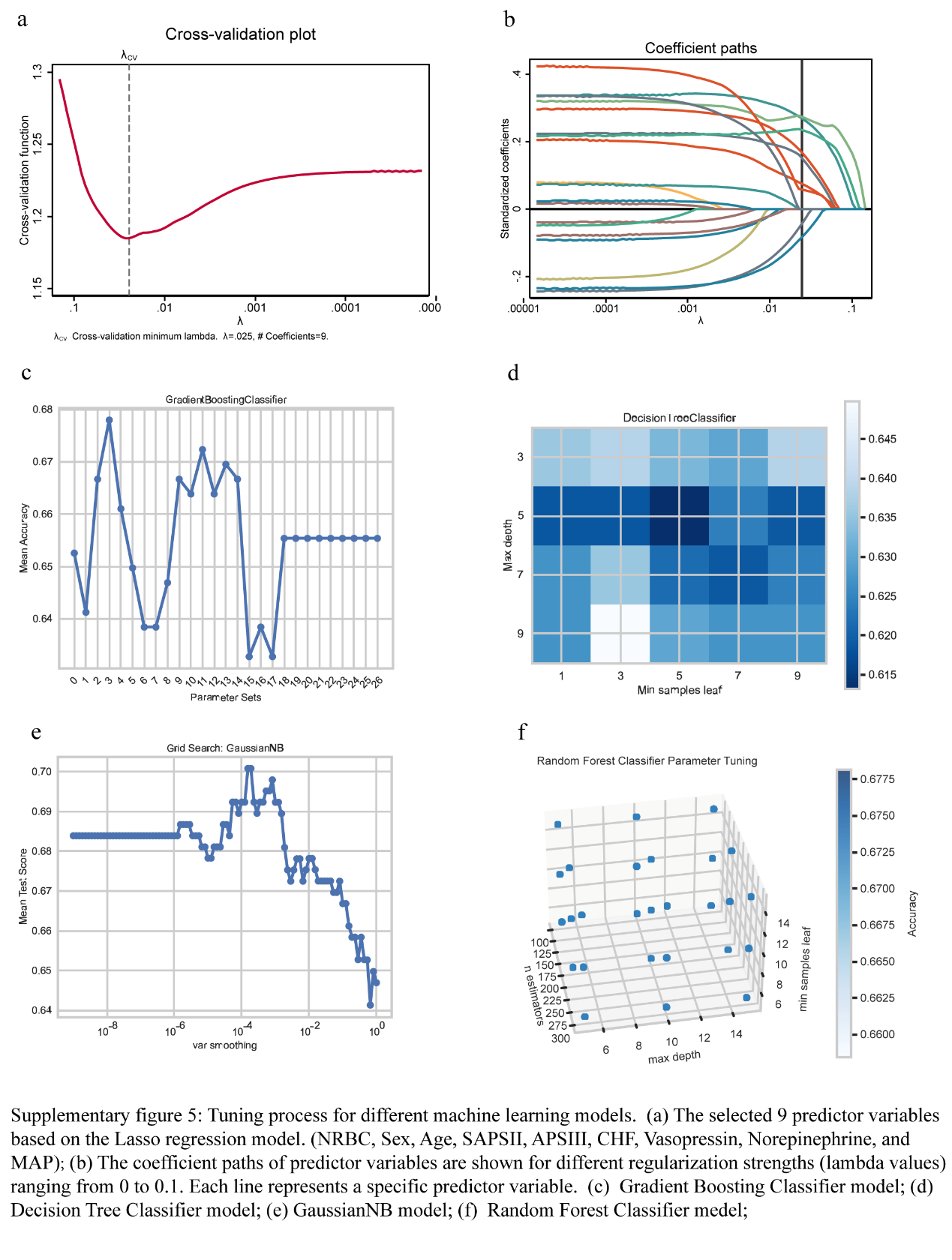


# Supplementing Figure 6


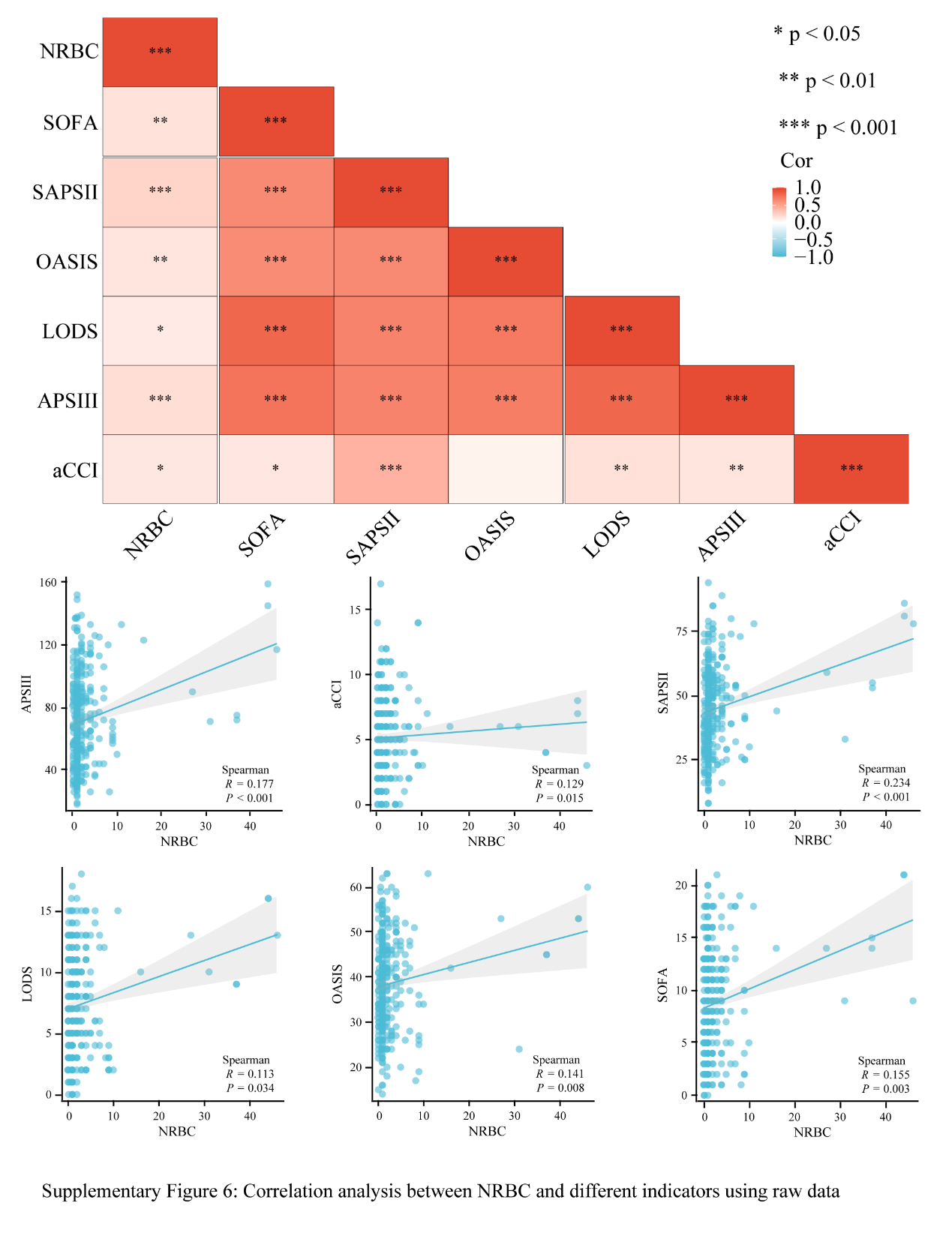


# Supplementing Figure 7


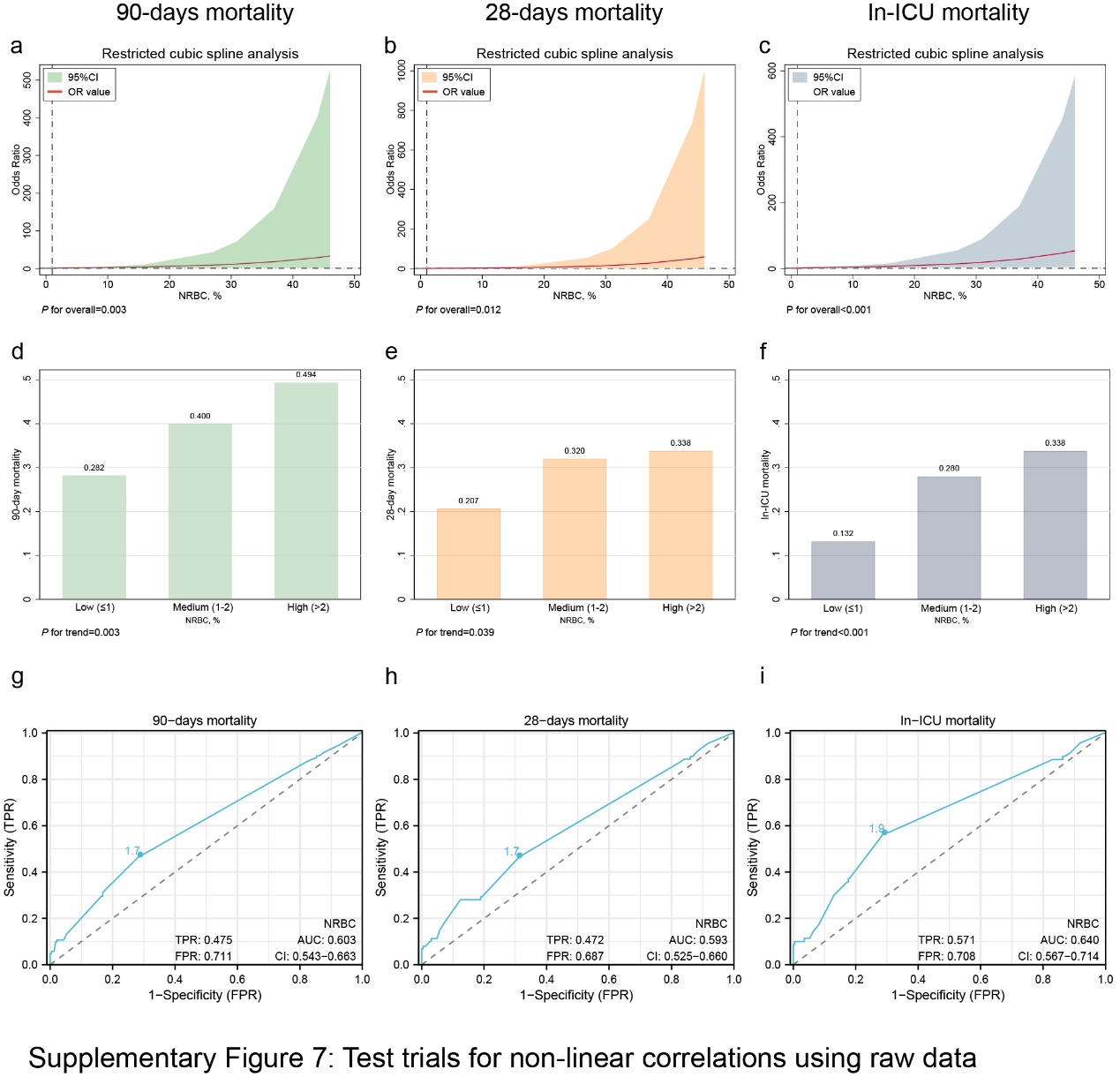


# Supplementing Figure 8


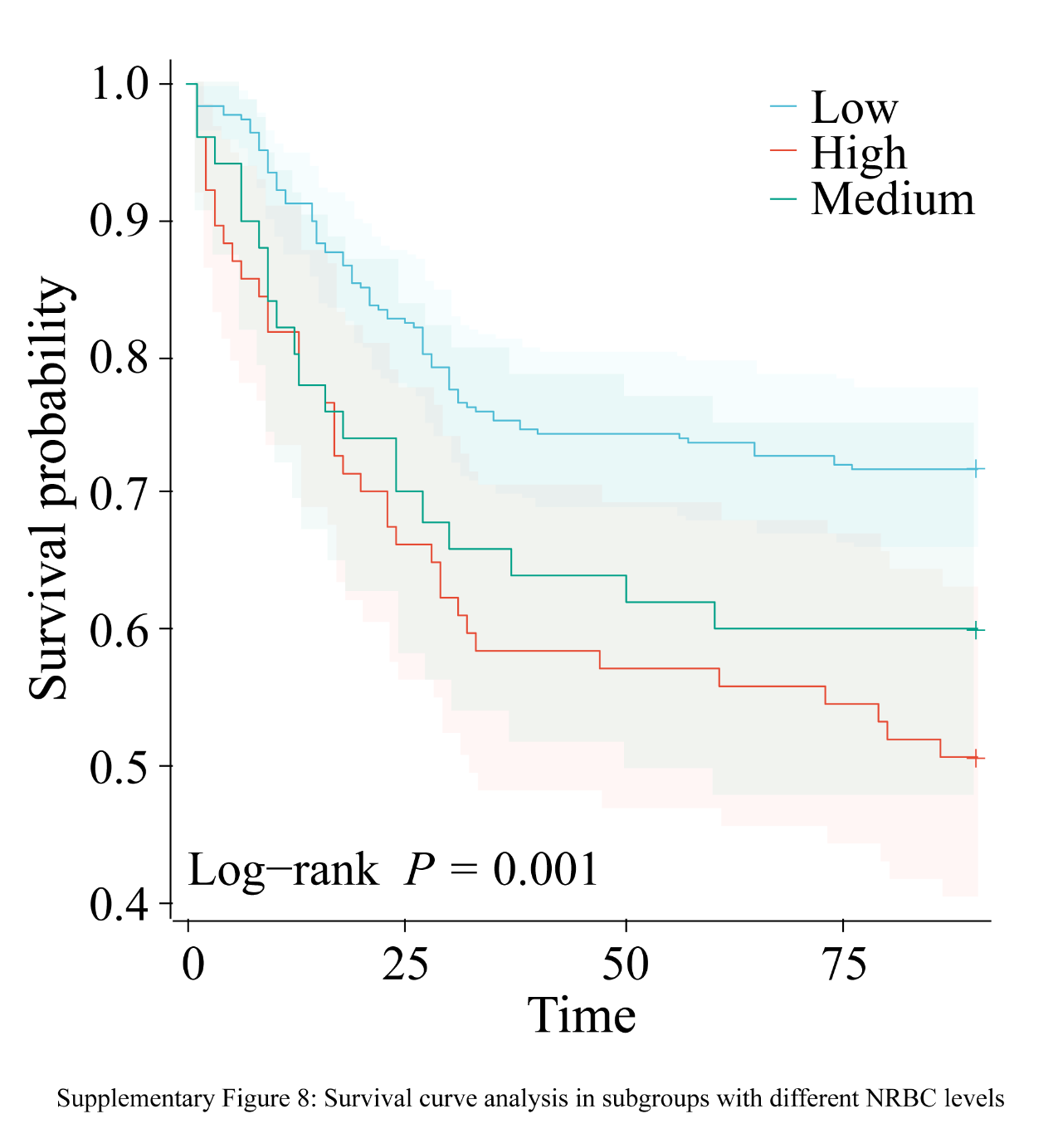


# Supplementing Table 1: ICD Codes for Acute Pancreatitis

| Variable | Code |
| --- | --- |
| ICD9 | 5770 |
| ICD10 | K8500 K8502 K8510 K8511 K8512 K8520 K8521 K8522 K8522 K8530 K8531 K8580 K8581 K8590 K8591 |

# Supplementing Table 2: Collinearity Test

| Variable | VIF | SQRT VIF | Tolerance | R-Squared |
| --- | --- | --- | --- | --- |
| NRBC | 1.14 | 1.07 | 0.88 | 0.12 |
| RRT | 1.29 | 1.14 | 0.77 | 0.23 |
| Sex | 1.12 | 1.06 | 0.89 | 0.11 |
| Race | 1.1 | 1.05 | 0.91 | 0.09 |
| Age | 1.43 | 1.19 | 0.70 | 0.30 |
| SOFA | 4.51 | 2.12 | 0.22 | 0.78 |
| SAPSII | 2.95 | 1.72 | 0.34 | 0.66 |
| OASIS | 2.59 | 1.61 | 0.39 | 0.61 |
| LODS | 5.67 | 2.38 | 0.18 | 0.82 |
| APSIII | 4.8 | 2.19 | 0.21 | 0.79 |
| diabetes | 1.17 | 1.08 | 0.85 | 0.15 |
| CHF | 1.32 | 1.15 | 0.76 | 0.24 |
| CPD | 1.09 | 1.05 | 0.91 | 0.09 |
| Renal disease | 1.3 | 1.14 | 0.77 | 0.23 |
| Vasopressin | 1.79 | 1.34 | 0.56 | 0.44 |
| Norepinephrine | 2.01 | 1.42 | 0.50 | 0.50 |
| Cerebrovascular disease | 1.09 | 1.04 | 0.92 | 0.08 |
| GCS | 2.36 | 1.53 | 0.42 | 0.58 |
| MAP | 1.27 | 1.13 | 0.79 | 0.21 |

Abbreviations: APS III, Acute Physiology Score III; CHF, Congestive Heart Failure; CPD, Chronic Pulmonary Disease; GCS, Glasgow Coma Scale; LODS, Logistic Organ Dysfunction System; MAP, Mean Arterial Pressure; NRBC, Nucleated Red blood Cells; OASIS, Oxford Acute Severity of Illness Score; RRT, renal replacement therapy; SAPS II, Simplified Acute Physiology Score II; SOFA, Sequential Organ Failure Assessment

# Supplementing Table 3: Univariate and multivariate GLM regression analyses using raw data

| Characteristics | Univariate analysis | |  | Multivariate analysis | |
| --- | --- | --- | --- | --- | --- |
|  | Odds Ratio (95% CI) | P value |  | Odds Ratio (95% CI) | P value |
| NRBC | 1.108 (1.030 - 1.192) | **0.006** |  | 1.100 (1.022 - 1.183) | **0.011** |
| Sex (Female *vs.* Male) | 0.803 (0.516 - 1.249) | 0.330 |  |  |  |
| Race (While *vs.* Non-while) | 1.042 (0.665 - 1.634) | 0.857 |  |  |  |
| Age (<60 *vs.* ≥60) | 2.805 (1.783 - 4.411) | **< 0.001** |  | 2.326 (1.322 - 4.093) | **0.003** |
| CHF (No *vs.* Yes) | 2.107 (1.296 - 3.424) | **0.003** |  | 1.816 (1.005 - 3.279) | **0.048** |
| CPD (No *vs.* Yes) | 1.286 (0.747 - 2.212) | 0.364 |  |  |  |
| Diabetes (No *vs.* Yes) | 1.099 (0.677 - 1.783) | 0.703 |  |  |  |
| Renal disease (No *vs.* Yes) | 2.019 (1.183 - 3.446) | **0.010** |  | 1.044 (0.553 - 1.970) | 0.895 |
| Cerebrovascular disease (No *vs.* Yes) | 0.670 (0.289 - 1.553) | 0.350 |  |  |  |
| Vasopressin (No *vs.* Yes) | 3.406 (2.134 - 5.437) | **< 0.001** |  | 1.732 (0.911 - 3.293) | 0.094 |
| Norepinephrine (No *vs.* Yes) | 2.809 (1.772 - 4.453) | **< 0.001** |  | 1.476 (0.749 - 2.912) | 0.261 |
| RRT (No *vs.* Yes) | 1.380 (0.636 - 2.991) | 0.415 |  |  |  |
| SOFA | 1.096 (1.044 - 1.150) | **< 0.001** |  | 0.984 (0.885 - 1.093) | 0.762 |
| SAPSII | 1.044 (1.028 - 1.061) | **< 0.001** |  | 1.026 (1.000 - 1.053) | 0.052 |
| OASIS | 1.042 (1.019 - 1.067) | **< 0.001** |  | 0.979 (0.942 - 1.019) | 0.299 |
| LODS | 1.129 (1.064 - 1.199) | **< 0.001** |  | 0.959 (0.835 - 1.101) | 0.553 |
| APSIII | 1.019 (1.011 - 1.027) | **< 0.001** |  | 1.012 (0.994 - 1.030) | 0.183 |
| GCS | 0.980 (0.930 - 1.032) | 0.442 |  |  |  |
| MAP | 0.991 (0.984 - 0.998) | **0.015** |  | 0.995 (0.987 - 1.003) | 0.185 |

Abbreviations: APS III, Acute Physiology Score III; CHF, Congestive Heart Failure; CPD, Chronic Pulmonary Disease; GCS, Glasgow Coma Scale; GLM, generalized linear model; LODS, Logistic Organ Dysfunction System; MAP, Mean Arterial Pressure; NRBC, Nucleated Red blood Cells; OASIS, Oxford Acute Severity of Illness Score; RRT, renal replacement therapy; SAPS II, Simplified Acute Physiology Score II; SOFA, Sequential Organ Failure Assessment

# Supplementing Table 4: Effect size of NRBC on mortality using raw data

| Model | 90-day mortality | 28-day mortality | In-ICU mortality |
| --- | --- | --- | --- |
| Crude model OR (95% CI) | 1.108 (1.030 - 1.192) | 1.103(1.036 - 1.176) | 1.115 (1.046 – 1.190) |
| *P*-value | **0.006** | **0.002** | **0.001** |
| Model1 OR (95% CI) | 1.132 (1.048 – 1.223) | 1.135 (1.058 - 1.218) | 1.132 (1.057 – 1.213) |
| *P*-value | **0.002** | **< 0.001** | **< 0.001** |
| Model2 OR (95% CI) | 1.133 (1.048 – 1.225) | 1.131 (1.055 - 1.213) | 1.128 (1.053 – 1.208) |
| *P*-value | **0.002** | **0.001** | **0.001** |
| Model3 OR (95% CI) | 1.100 (1.022 - 1.183) | 1.098 (1.028 - 1.174) | 1.102 (1.016 - 1.196) |
| *P*-value | **0.011** | **0.005** | **0.019** |

Note: Crude model: unadjusted for confounding factors; Model1: adjusted for sex and age; Model2: adjusted for sex, race, and age; Model3: adjusted for age, CHF, Renal disease, Vasopressin, Norepinephrine, SOFA, SAPSII, OASIS, LODS, APSIII, and MAP.
